# Another Journal on *Nanomaterials*?

**DOI:** 10.3390/nano1010001

**Published:** 2010-11-04

**Authors:** Thomas Nann

**Affiliations:** *Founding Editor-in-Chief of Nanomaterials*, University of South Australia, Ian Wark Research Institute, Mawson Lakes Blvd, Adelaide SA 5095, Australia; E-Mail: thomas.nann@unisa.edu.au; Tel.: +61-8-8302-5369; Fax: +61-8-8302-3683

It is my great pleasure to welcome you to *Nanomaterials*, a new open access journal, which is dedicated to the fabrication, characterization, functionalization, modeling and application of nanomaterials. In answer to the title question, I would like to (mis)quote one of my favourite pieces of literature: *I “[We] hold these truths to be self-evident, that all men … are endowed … with certain unalienable rights, that among these are …” free access to information and education*. The prime goal of *Nanomaterials* is to publish first-class, original research articles under an open access policy with minimal fees for the authors. The quality of the published articles will be assured by a fast yet rigorous peer-review process.

Since *Nanomaterials* is published as an online journal, there are no page restrictions in place. However, we distinguish between different types of publications: *Short Communications* contain key elements of a larger research project or preliminary results, and will be published rapidly. *Full Articles* are comprehensive reports on original research of the highest quality. Finally, *Ideas* will discuss novel and thought-provoking scientific ideas and concepts that do not necessarily have to be fully proven (yet). In our *Book Review* section, we will publish helpful reviews on new nanomaterial related books. Again, the overarching aim of *Nanomaterials* is to publish excellent, peer-reviewed articles.

Nanomaterials are characterized by their mesoscopic properties (properties arising from materials having size dimensions in the mesoscopic scale—*i.e.*, quantum confinement effects). These properties can be found in very diverse materials and applied in fields ranging from nanomedicine to laser physics. Consequently, the scope of *Nanomaterials* embraces many scientific disciplines, as long as nanomaterials are a crucial part of the work. More specifically, *Nanomaterials* publishes articles in the areas of:

- Preparation of nanomaterials
- Properties of nanomaterials (including characterization)
- Modification (derivatization) of nanomaterials
- New applications of nanomaterials (with the restriction that the manuscript must contain at least one novel aspect about the nanomaterial itself, e.g., manuscripts where commercial Quantum Dots have been employed in a bioanalytical application will not be accepted)
- Modeling of nanomaterials
- Bio-nanomaterials
- Nanomedicine

I hope that this exciting project sparks your interest, that you enjoy reading *Nanomaterials*, and we are looking forward to receiving some of your very best manuscripts for publication in this journal!

